# Harnessing oscillatory fluid behaviour to improve debris wash-out in ureteroscopy

**DOI:** 10.3389/fruro.2023.1182919

**Published:** 2023-08-30

**Authors:** Harry C. A. Reynolds, Ben W. Turney, Sarah L. Waters, Derek E. Moulton

**Affiliations:** ^1^ Mathematical Institute, University of Oxford, Oxford, United Kingdom; ^2^ Nuffield Department of Surgical Sciences, John Radcliffe Hospital, University of Oxford, Oxford, United Kingdom

**Keywords:** kidney stone, cavity flow, mathematical model, fluid irrigation, vorticity

## Abstract

In ureteroscopy, a common method for kidney stone removal, a ureteroscope is inserted into the patient’s kidney, through which working tools such as a laser are inserted. During the procedure, the renal space proximal to the scope tip is irrigated with fluid in order to clear stone particles and debris. However, even with continual fluid flow into and out of the kidney, stone dust may become trapped in vortical structures, significantly impairing the operating clinician’s field of view. Key to overcoming this challenge is a clear understanding of the flow patterns within an irrigated kidney calyx, and a modelling framework that enables to interrogate how different flow conditions impact on the wash-out time of debris. Previous theoretical studies have uncovered the interplay between fluid structure, in particular the presence of vortical regions, and dust washout, but only in a regime of steady inlet flow conditions. In this paper we model a kidney calyx in an idealised 2D cavity geometry, in which we investigate the presence and potential disturbance of vortical structures due to an oscillatory inlet condition, and the impact on dust washout, modelled as a passive tracer in the flow. By varying the flow amplitude and frequency at the inlet, we uncover a delicate relationship with vortex size and vortex disturbance, and we demonstrate the potential for significant decrease in wash-out time with low-frequency high-amplitude conditions. We then compare this result to the commonly used practice of flushing, a discrete and temporary increase in flow, and we also demonstrate the qualitative robustness of our findings to changes in cavity geometry.

## 1 Introduction

In the United States, approximately one in eleven people are affected by kidney stones (1). The disease is typically more common in males, with around 12% of men and 5% of women developing a kidney stone at some point during their lifetime (2). Stone disease is a great burden on national health resources, with the financial cost in the USA estimated to exceed five billion dollars by 2030 (3). Even once the disease is treated, the risk of recurrence is around 40% after 5 years, rising to 75% after 20 years (4). These are just some factors that motivate the need for efficient and effective treatment of kidney stones.

While smaller stones are usually left to pass naturally out of the body in the urine, larger ones can remain stuck within the kidney calyx. For these larger stones, an approach which is quickly becoming the preferred method for their removal is ureteroscopy (5). The procedure involves passing a flexible medical instrument, known as a ureteroscope, through the urinary system to gain access to the kidney. The ureteroscope is hollow along its length, creating a working channel through which working tools such as wires, baskets, and laser fibres can be passed to access and remove the kidney stones. Once a clinician has access, a high-powered laser is fired at the stone to reduce it to dust, or break it into small fragments which may be removed by grabbing them using a wire basket. Modern scopes are also fitted with a light source and a camera at their tip, allowing urologists to visualise the urinary system and locate the stones. Ureteroscopy requires constant fluid irrigation to wash away stone dust created during the laser treatment, enabling the clinician to have a clear field of view. The fluid irrigation also keeps the urinary tract dilated, providing better scope manoeuvrability during the procedure.

Traditionally, fluid irrigation is achieved by hanging a bag of saline solution above the level of the scope, creating a gravity-driven flow. The irrigation fluid flows from the bag into the scope, then down the length of the working channel before exiting out of the tip of the scope into the kidney. The fluid then flows out of the patient in the space between the outside of the scope and the ureter wall. Often an access sheath is inserted within the ureter to provide ureteral dilation, creating a channel for the scope to pass through. Understanding the relation between fluid flow within the cavities of the kidney and stone dust removal is key to determining fluid irrigation protocols for enhancing the efficiency of ureteroscopy.

From a fluid mechanics perspective, the key ingredients in the ureteroscopy system are pipe flow into a cavity, an outlet pipe flow with the exit located on the same side of the cavity as the inlet, and a diffusive dust that advects with the flow. A related but perhaps simpler system is pipe flow with a sudden expansion in the radius of the pipe, which provides one of the classic examples of separated flows. Durst et al. (6) studied pipe expansion at low Reynolds number, where the Reynolds number characterises the relative effects of inertia and fluid viscosity. Via a numerical finite volume method and supported by experiments, they showed that recirculation zones will form close to the cavity entrance. As the Reynolds number increases, the zones increase in size and asymmetry in the flow occurs. This asymmetry was further explored in a numerical study (7), finding a critical Reynolds number at which a symmetry-breaking bifurcation occurs. Mizushima et al. (8) showed that the addition of a downstream contraction after the cavity can re-stabilise the asymmetry in the flow, work that was later extended to two outlets (9).

The studies above highlight the presence of flow asymmetry and recirculation zones, features that play a strong role in the removal of debris in the fluid. Efficient debris removal is important in many different fields, such as in cleaning and decontamination. Min et al. (10) considered mass transfer in a laminar flow downstream of a backward facing step, for varying Reynolds and Schmidt numbers, where the Schmidt number is the ratio of kinematic viscosity to diffusivity. They found that mass accumulates in recirculation zones located near the step. The interplay between advective and diffusive effects was considered, and it was shown that an increase in Schmidt number strongly decreased the rate of contaminant removal. Within the biomedical field, accumulation of debris is often undesirable and so the reduction of recirculation zones to improve mass transfer is a topic of great interest. Previous studies in this direction include (11), which characterised the flow patterns within stented ureters and showed that recirculation zones often occur. In (12), Jimenez´ and Davies similarly modelled blood flow through stented arteries and found that the recirculation zones act as sites for the accumulation of inflammatory elements. Possible redesigns of the stents were shown to reduce recirculation zones and thus improve wash-out. A related study with particular relevance for the present work is that of Cooloa and Caro (13), in which the authors investigated steady and unsteady flows in stented arteries and showed that flow pulsatility plays an important role in disturbing vortices.

Several recent papers have specifically modelled the fluid mechanics and dust removal properties of the ureteroscopy system. Some of these studies have focused on the properties of the pipe flow and how it relates to the shape of the cross-section and kidney pressure (14–16). More relevant to the present research are the papers (14) and (17), which studied fluid flow and advection-diffusion of a passive tracer representing stone dust within a 2D representation of the renal cavity. In (14), the presence and size of vortical structures was characterised as a function of Reynolds number, with numerical results validated by particle image velocimetry experiments, and the negative impact of vortical structures on debris clearance was quantified. Williams et al. (17) extended this work by allowing the shape of the inlet channel to vary, seeking an optimal shape for the rapid washout of the passive tracer.

Key to previous work on debris washout is the conceptual point that washout time is strongly linked to the presence of vortical structures in which debris may become trapped. It is worth noting that the studies by Williams et al. above utilised a fixed upstream pressure, such that the fluid boundary condition at the inlet to the cavity was constant in time. In this paper, we relax this assumption and consider a time-varying inlet condition for the flow. A strong motivating factor for the present research is a recent advancement in the field of ureteroscopy: the traditional fluid delivery method of an upstream hydrostatic pressure generated by a hanging bag of saline may be replaced by an alternative method involving a peristaltic pump that drives the fluid within a fully controllable fluid management system. In contrast to gravity-driven flow, this delivery method enables a much finer control over the flow rate, in particular giving the ability to modulate the flow rate as a function of time. This technology, combined with previous studies such as (13) that have demonstrated the potential to disturb vortices using flow pulsatility, motivates the key question we seek to answer in this paper: can a time-varying inlet be utilised to enhance vortical disruption and thus reduce the washout time of debris?

Following the above works by Williams et al., we model the kidney calyx as a two-dimensional rectangular domain, with kidney stone dust modelled as a passive tracer transported within and eventually out of the cavity by the combined effects of diffusion and advection. We consider the scenario in which an initially steady flow into the cavity is modulated by a sinusoidal component. The model is outlined in Section 2. We characterise in Section 3 the presence and size of vortices, their disturbance due to oscillatory inlet, and the impact on debris washout, within the parameter space of the properties of the inlet flow (base value, amplitude, and frequency), and the diffusivity of the tracer. Within this 4 dimensional space we demonstrate that oscillations can indeed be harnessed in order to reduce the washout time, uncovering in the process a fine mechanistic balance between the frequency and amplitude of the imposed flow pulsatility and the size and disruption of vortical regions. We then compare in Section 3.3 the reduction in washout under the oscillatory regime to a simulation of a typical industry method termed flushing – an instantaneous jump in flow rate from one steady value to another for a usually brief period of time. In Section 3.4 we demonstrate the robustness of our conclusions to different cavity geometries. Conclusions are given in Section 4.

## 2 Problem description

**Figure 1:**
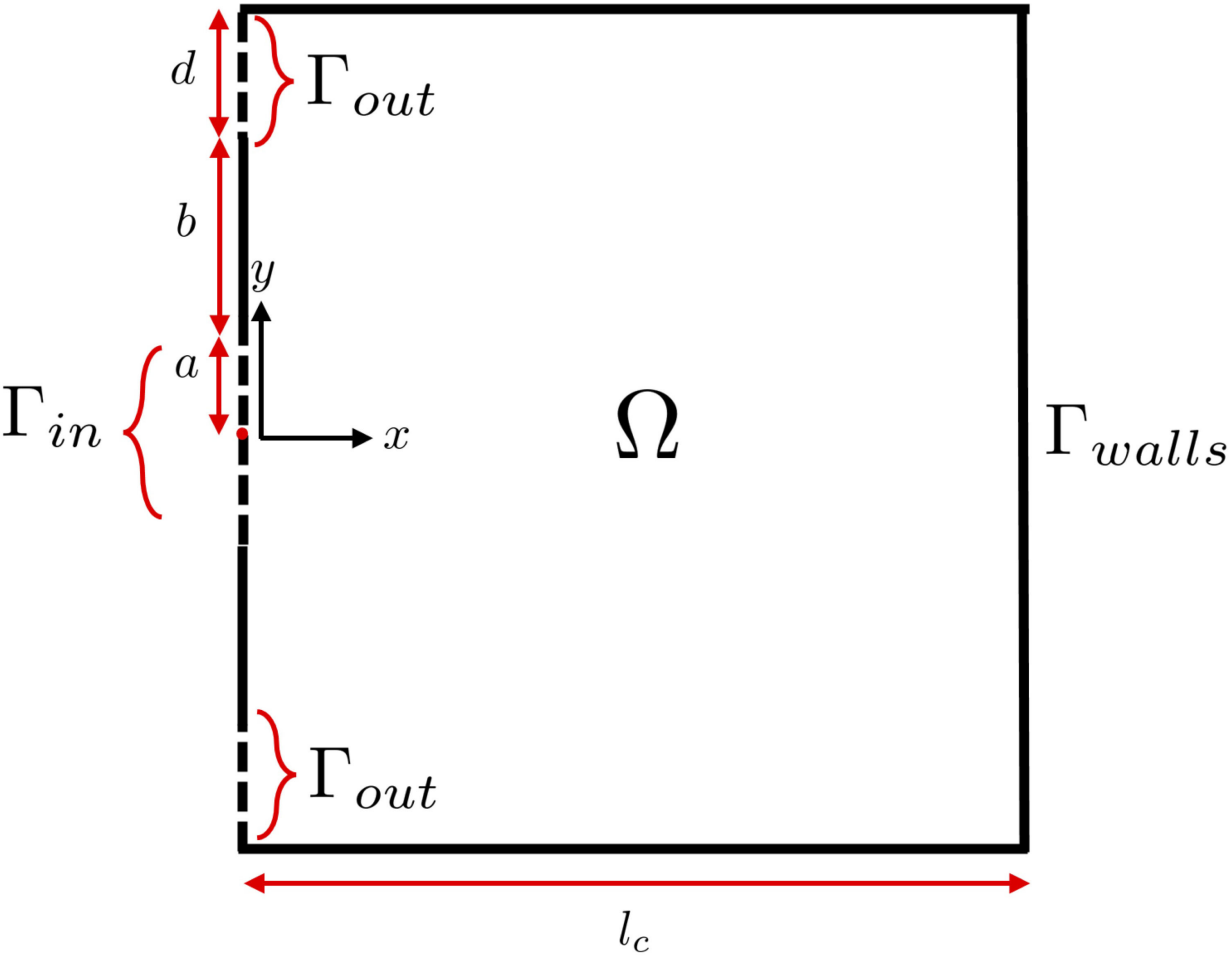
Kidney cavity domain sketch with domain Ω of length $l_{c}$ and width 2(a+b+d) , with boundary Γ comprising of inlet ${\text{Γ}}_{in}$ , outlets ${\text{Γ}}_{out}$ , and impermeable boundaries ${\text{Γ}}_{wall}$ .

We model a kidney calyx as a two-dimensional, rectangular cavity described in Cartesian coordinates $x^{*}=\left(x^{*},y^{*}\right)$ with corresponding coordinate directions $\left(e_{x},e_{y}\right)$ . The cavity domain Ω has length $l_{c}$ and width 2(a+b+d) , with boundary Γ comprising of inlet, outlets, and impermeable boundaries. The fluid enters the cavity through a single inlet of width 2a on the left-hand side of the domain, denoted ${\text{Γ}}_{in}$ , and exits through two outlets each of width d , denoted ${\text{Γ}}_{out}$ , located on the same side, above and below the inlet (**Figure 1**). The remaining boundaries are denoted by ${\text{Γ}}_{wall}$ , representing the walls of the cavity which are assumed to be impermeable to both fluid and dust.

The fluid has velocity ${\text{u}}^{*}=\left(u^{*}\left(x^{*},y^{*},t^{*}\right),\;v^{*}\left(x^{*},y^{*},t^{*}\right)\right)$ and pressure $p^{*}\left(x^{*},y^{*},t^{*}\right)$ , where $t^{*}$ denotes time, and the dust concentration is given by $c^{*}\left(x^{*},y^{*},t^{*}\right)$ . The fluid has density $\rho^{*}$ and dynamic viscosity $\mu^{*}$ , and the kidney stone dust has diffusion coefficient $D^{*}$ . Corresponding to the assumption of impermeable walls, we impose zero velocity on the cavity walls. We impose at the inlet the following velocity profile


(1) $$u^{*}=\left({\bar{U}}^{*}+{\hat{U}}^{*}\text{cos }(\omega^{*}t^{*}\right))\left(1-\frac{y^{*2}}{a^{2}}\right),\;v^{*}=0,$$

where $\omega^{*}$ is the frequency of the oscillatory component. The outlet of the cavity connects to the access sheath, through which fluid exits out of the ureteroscope into atmospheric conditions with pressure $p_{\text{atm}}^{*}$ . The natural boundary condition at the end of the access sheath is continuity of stress. For simplicity, we neglect this component of the flow, which is simple pipe flow, and impose a stress free condition on the fluid at the exit of the cavity. Justification for this simplification is given in (18), in which it is shown that explicitly including the access sheath as an exit channel has only a very small quantitative and no qualitative effect on flow properties.

Motivated by the clinical scenario in which an oscillatory component is added to an already existing steady flow, as an initial condition for the fluid we solve the corresponding steady problem with inlet condition


(2) $$u^{*}={\bar{U}}^{*}\left(1-\frac{y^{*2}}{a^{2}}\right),\;v^{*}=0,$$

and use the computed flow solution as an initial condition for the unsteady problem (**Appendix 7**).

We assume zero flux of dust at the inlet and cavity walls, and zero diffusive flux at the outlet. Hence, dust can exit the cavity via advection through either outlet. We assume that initially the dust occupies the entire cavity domain with a uniform concentration $C^{*}$ .

### 2.1 Dimensionless governing equations

We non-dimensionalise as follows:


(3) $$x^{*}=ax\;,\;{\text{u}}^{*}={\bar{U}}^{*}\text{u },\;p^{*}=\frac{\mu^{*}{\bar{U}}^{*}}{a}p-p_{atm,\;}^{*}t^{*}\;=\frac{a}{{\bar{U}}^{*}}t\;,\;c^{*}=C^{*}c.$$

The non-dimensional, time-dependent Navier-Stokes equations governing the fluid are given by


(4) $$Re\left(\frac{\partial \text{u}}{\partial t}+\left(\text{u}\cdot \nabla \right)\text{u}\right)=-\nabla p+\nabla^{2}\text{u },$$

(5) ∇·u=0 .

where $Re=\rho^{*}{\bar{U}}^{*}a/\mu^{*}$ is the Reynolds number. The dimensionless inlet flow profile is


(6) $$u=\left(\left(1+\frac{\hat{U}}{Re}\text{cos }\left(\frac{\omega }{Re}t\right)\right)\left(1-y^{2}\right),0\right){\text{ on Γ}}_{in}\;,$$

where $\hat{U}=\rho^{*}{\hat{U}}^{*}a/\mu^{*}$ is the dimensionless amplitude and $\omega =\rho^{*}\omega^{*}a^{2}/\mu$ is the dimensionless frequency. To prevent backflow, we restrict attention to the regime $\hat{U}<Re$ . At the outlet boundaries we prescribe zero stress such that


(7) $$\sigma \cdot \text{n}=0{\text{ on Γ}}_{out}\;,$$

where ***n*** is a normal vector to the boundary. We impose no-slip and zero normal velocity on all impermeable walls, given by


(8) $$\text{u}=\left(0,\;0\right){\text{ on Γ}}_{walls}\;.$$

The initial condition for the fluid is given by


(9) $$\text{u}\left(x,0\right)={\text{u}}_{steady}\left(x\right)\;,$$

where ${\text{u}}_{steady}\left(x\right)$ is the solution to the steady system, as described above.

Dust transport within the cavity is governed by the advection-diffusion equation, given in non-dimensional form by


(10) $$\frac{\partial c}{\partial t}=\frac{1}{ReSc}\nabla^{2}c-\text{u}\cdot \nabla c\;,$$

where $Sc=\mu^{*}/\rho^{*}D^{*}$ is the Schmidt number. Note that the dust is coupled to the fluid via the advection term (second term on the right hand side), but that the fluid flow is not affected by the dust^1^. The dust is subject to the boundary conditions


(11) $$\nabla c\cdot \text{n}=0{\text{ on Γ}}_{walls}\;,$$

(12) $$\text{J}\cdot \text{n}=0{\text{ on Γ}}_{in}\;,$$

(13) $$\nabla c\cdot \text{n}=0{\text{ on Γ}}_{out}\;,$$

where $\text{J}={\left(ReSc\right)}^{-1}\nabla c-\text{u}c$ is the total (dimensionless) flux, and initial condition


(14) c(x,0)=1  on  Ω .

For given geometry, note that the system is characterised by four non-dimensional parameters: $Re,Sc,\omega ,\hat{U}$ . A primary aim is to determine the impact these parameters have on the fluid behaviour and dust transport in the system.

### 2.2 Metrics

In order to analyse and quantify system behaviour, we now define three relevant metrics. First, in order to identify recirculation zones in the fluid, we follow (21) and define the metric Θ(t) as


(15) $$\text{Θ}\left(t\right)=\iint_{\Omega }\theta \;d\text{Ω where }\theta :=\text{max}\left(0,\text{ det}\nabla \text{u}\right)\;.$$

This metric quantifies vortical regions in the flow by measuring the magnitude of the vorticity over time. For a sinusoidally varying inlet condition, Θ will take the approximate form $\text{Θ}\left(t\right)={\text{Θ}}_{avg}+{\text{Θ}}_{amp}\text{cos }(\omega t)$ where $ta_{avg}$ gives a measure of the average size of vortical regions and ${\text{Θ}}_{amp}\leq {\text{Θ}}_{avg}$ provides a measure for vortical disruption.

In terms of dust, we define two further metrics. The total concentration of dust remaining in the domain at time t is given by the metric


(16) $$\gamma \left(t\right)=\iint_{\text{Ω}}c\left(x,t\right)\;d\text{Ω }.$$

To measure dust washout, we follow (17) in defining the metric $T_{90}$ implicitly via


(17) $$\frac{\iint_{\Omega }\left(c\left(x,\;0\right)-c\left(x,\;T_{90}\right)\right)\;d\Omega }{\iint_{\Omega }c\left(x,\;0\right)\;d\Omega }=0.9\;$$

which measures the time taken for 90% of dust to leave the cavity. To allow in principle comparison across varying Reynolds numbers, we normalise the above metrics with respect to the mean velocity scale, and present in what follows ΘRe and $T_{90}/Re$ .

### 2.3 Numerical details

We solve the system numerically via a finite element formulation. The problem is implemented using the open source finite element library Firedrake (22). To solve the fluid equations, we adapt a stationary incompressible Navier-Stokes solver with Reynolds robust pre-conditioner (23) in order to solve the unsteady problem. For the advection-diffusion equation, we transform equation (10) into weak form. The fluid solution is given as an input to the weak form of the advection-diffusion equation, which we then solve numerically via the inbuilt Firedrake solver with a backward Euler scheme for the time derivative. The cavity mesh structure is generated via the open source finite element mesh generator software Gmsh (24). For further details, see (25).

### 2.4 Steady solution behaviour

Before considering the impact of oscillatory inlet flow, we review the fluid structure under steady inlet flow in order to select an appropriate Reynolds number. Williams et al. (26) showed that the fluid solution in the cavity undergoes a saddle bifurcation at a critical Reynolds number. Below this critical point a single, symmetrical solution can be seen. In this regime, vortical regions are small and thus debris washout is not hindered by vortical entrapment. Above the critical value, the symmetrical solution becomes unstable, and two stable asymmetric solutions appear. This is reminiscent of the symmetry breaking bifurcation with increasing Reynolds number that is observed with two jets interacting in a channel (19). These two solutions are reflections of one another in the line y=0 . They display a large single vortex in the centre of the cavity, where either the upper or lower outlet is the main exit point for the fluid. As the Reynolds number increases, the vortex within the cavity grows larger, trapping more dust within it and thus increasing the associated wash-out times. It is in this regime that we seek to enhance washout by vortical disruption.

For the geometric parameters we consider here, the critical Reynolds number is *Re* ≈ 20. An investigation of relevant physical parameters for the ureteroscopy system (see **Supplementary Material section 2**) suggests a range from about *Re* ∼ 200 to *Re* ∼ 2000 based on inlet velocity, though those values are only strictly applicable for 3D flow. An experimental setup designed to mimic 2D flow in a rectangular cavity with the same geometry as we consider here used a Reynolds number *Re* ∼ 40 (26). Note as the Reynolds number increases, the size of the central vortex also increases (18). In an advection-dominated flow, the larger the vortex, the longer it will take for dust particles to be removed, particularly in regimes where vortex disruption is minimal. There is a very high associated computational cost with this; as explored in **Supplementary Material section 2**, dust clearance takes on the order of 7 hours to compute for *Re* ∼ 100. To maintain a more tractable computational system, here we fix *Re* = 50, a value at which we retain the property of a large central vortex in which dust may become trapped, but not so large that computation of washout becomes overly expensive.

## 3 Results

### 3.1 Vortical disruption

**Figure 2:**
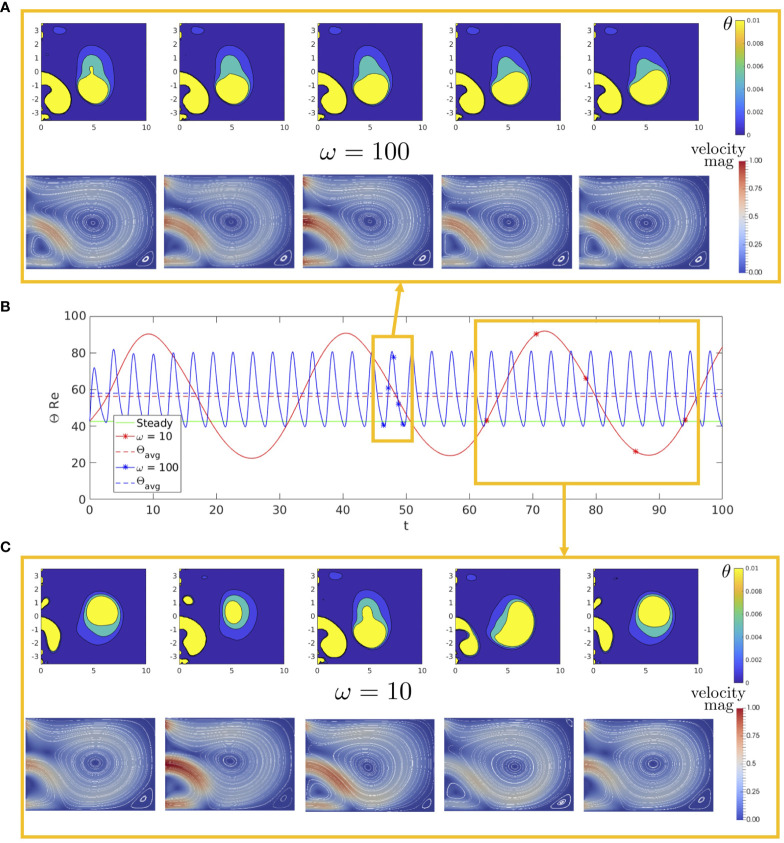
**(B)** Θ Re over time for ω=10 , ω=100 , and a steady inlet condition, with the average value of the oscillatory cases given as dashed lines. Alongside this we show snapshots over a single period of the fluid velocity magnitude with streamlines and cavity circulation θ for both **(A)** ω=100 and **(C)** ω=10 , with Re=50 and $\hat{U}=20$ for each.

We next analyse the two parameters of the fluid system that characterise the inlet flow. We begin by fixing the inlet amplitude to be $\hat{U}/Re=.04$ and varying the inlet frequency ω . In **Figure 2**, we plot ΘRe over time for low frequency ω=10 , high frequency ω=100 , and no oscillations ω=0 . Alongside this we present snapshots of the fluid velocity magnitude with streamlines and spatial variation of θ (Eqn 15) over a single period, for both low and high frequencies. We begin by comparing the steady Θ value, given in green, to the ${\text{Θ}}_{avg}$ value of both the time-dependent solutions. We find a higher ${\text{Θ}}_{avg}$ value in both unsteady inlet cases, suggesting that an oscillating inlet causes the system on average to have a larger vortex measure than the steady system, and we also see that the average vortex size is largely independent of frequency. The high frequency solution snapshots reveal that the flow patterns within the cavity do not vary greatly over its period, with the centre of the vortex staying fairly stationary over time. Comparing **Figures 2A–C**, we observe that in the low frequency case, there is a larger variation in the vortex structure over a single period, with the centre of the vortex moving more between snap shots. The wider variation in vortices is quantified by the larger amplitude of Θ . We conclude that of these three cases, oscillating the inlet at a low frequency causes the largest disturbance to the vortices within the cavity.

**Figure 3:**
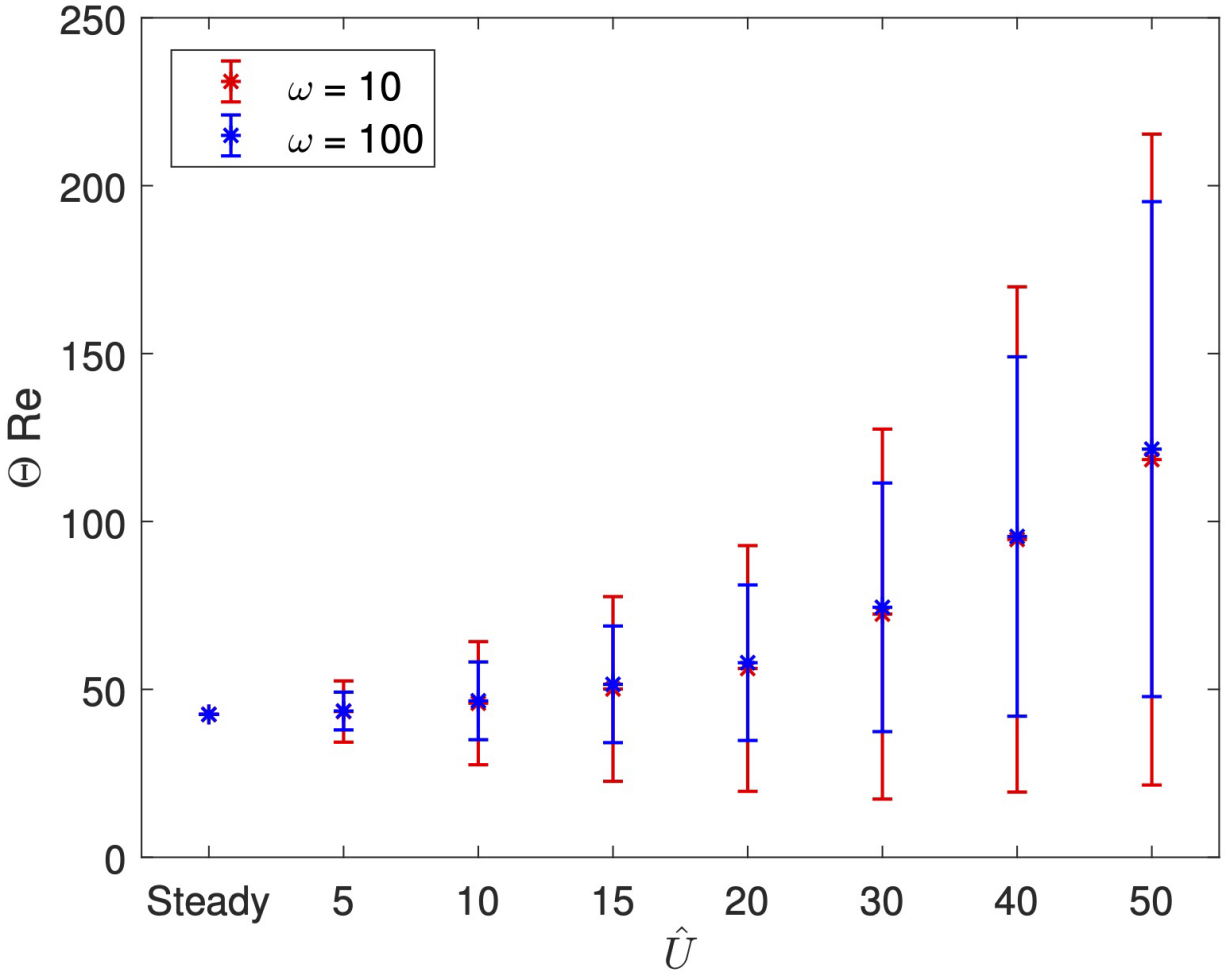
Θ Re for increasing $\hat{U}$ , with stars denoting $\Theta_{avg}Re$ and bars denoting $\Theta_{amp}Re$ , for Re=50 and ω=10,100 .

We now consider whether this observation holds for different amplitudes of the inlet flow. In **Figure 3**, we present ΘRe for increasing $\hat{U}$ , with stars denoting ${\text{Θ}}_{avg}Re$ and bars denoting ${\text{Θ}}_{amp}Re$ , in both the low ( ω=10 ) and high ( ω=100 ) frequency cases. As the amplitude increases, the average measure of the vortices ${\text{Θ}}_{avg}Re$ increases, at a similar rate for both frequencies, indicating that increasing amplitudes leads to an increase in the size of vortical regions when compared to the steady system. We also see that the variation in vortical structures over time increases as $\hat{U}$ increases, as shown by growing size of the bars. Again, we find little difference in ${\text{Θ}}_{avg}Re$ between the two frequencies, but that the lower frequency inlet causes a larger variation in vortices than the higher frequency.

The above analysis has shown that the low frequency, high amplitude oscillation has a greater effect in disturbing the vortices compared to high frequency. We anticipate, and demonstrate explicitly below, that increased vortical disruption will lead to decreased wash-out time. On the other hand, a high amplitude oscillation also creates a larger vortical region, i.e. a larger region where dust can become trapped, independent of frequency. The question remains whether enhanced vortical disturbance is sufficient to offset the increased vortical size and thus reduce dust wash-out times. To interrogate this, we next incorporate the transport of dust within the cavity via solving the advection-diffusion equation (10).

### 3.2 Dust clearance

In our modelling framework, dust transport occurs due to a combination of advection and diffusion. Diffusion is characterised by the Schmidt number, as defined in (10). For ureteroscopy, an examination of the properties of typical stone dust (see **Supplementary Material section 2**) suggests that the Schmidt number is *O*(10^7^) or higher. In this range diffusion plays almost no role at all, and clearance of dust from the kidney occurs due almost entirely to advection out of the cavity. In the case of vortical structures with closed streamlines, however, dust will remain trapped, as advection is not sufficient to transport dust out of these structures. Clearance of such dust requires an alternative mechanism. The premise of our work is to consider oscillatory flow, which has the potential to disrupt the vortices and enable dust particles to escape. To examine this hypothesis in a theoretical model, for comparative purposes we require a means of quantifying washout that is tractable both in cases with and without significant vortical disruption. We have followed here previous work in using the metric *T*_90_, the time for 90% of the dust to clear the cavity. The issue with this is that in cases of minimal vortical disruption, this time will only be reached on the diffusive timescale, which is orders of magnitude higher than the advective timescale. In particular, computing *T*_90_ for *Sc* only as high as 100 in the case of high frequency inlet (*ω* = 100) is very computationally expensive (**Supplementary Material section 2, Table 3**), as the flow and concentration must be continually resolved on the oscillatory timescale, but integrated forward in time on the diffusive timescale. In order to make progress and examine the impact of the frequency and amplitude of inlet oscillations on the washout metric *T*_90_, we thus fix *Sc* = 10; this value allows for efficient computation even in cases of minimal vortical disruption, and thus enables for qualitative comparison of oscillations on washout. The trade-off is that for some parameters we vastly underestimate the washout time, a point we return to in the discussion.

**Figure 4:**
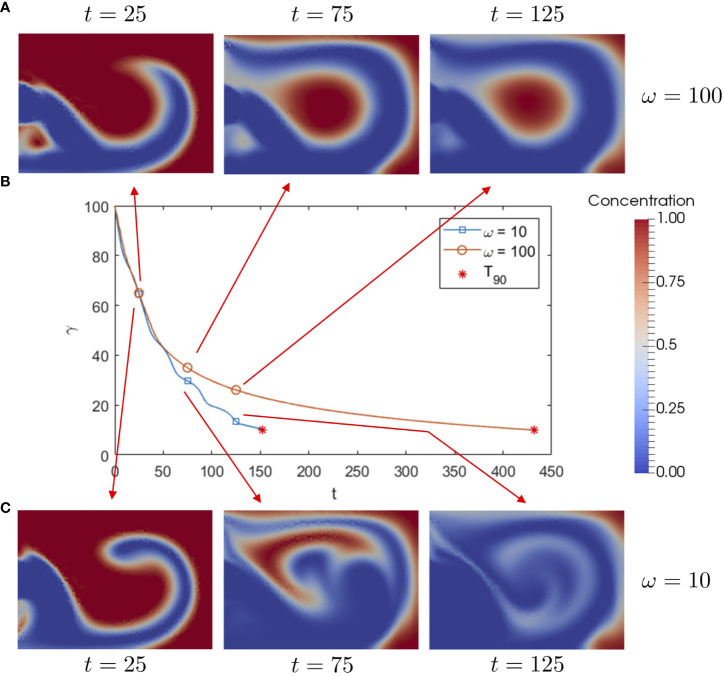
**(B)** Total dust concentration γ over time for ω=100 and ω=10 , stopping when the $T_{90}$ value is reached, along with snapshots of dust concentration c in the cavity for **(A)** ω=100 and **(C)** ω=10 , for Re=50 , Sc=10 and $\hat{U}=20$ .

We begin our analysis of washout by examining how a change in frequency impacts dust transport. In **Figure 4**, we plot total concentration γ over time for ω=10 and ω=100 , alongside snapshots of the dust concentration within the cavity, for $\hat{U}=20$ . Initially, the dust contained within non-closed streamlines is advected out of the cavity through both the upper and lower outlets. In the high frequency case, the remaining dust becomes trapped within the closed streamlines of the large central vortex and leaves on a slow diffusive timescale. The slow diffusion process leads to a high $T_{90}$ value. Similar results are seen in the steady system as shown in (26), suggesting that dust transport is comparable for both a steady parabolic inlet and a high frequency oscillatory inlet. In the low frequency case, the resulting wash-out time is significantly reduced. The improvement is evidently due to the increased disruption of the central vortex, enabling trapped dust to escape the vortex. We note that in both cases, a small amount of dust remains trapped in the back corners of the cavity, as this region remains relatively unaffected by the oscillatory flow.

**Figure 5:**
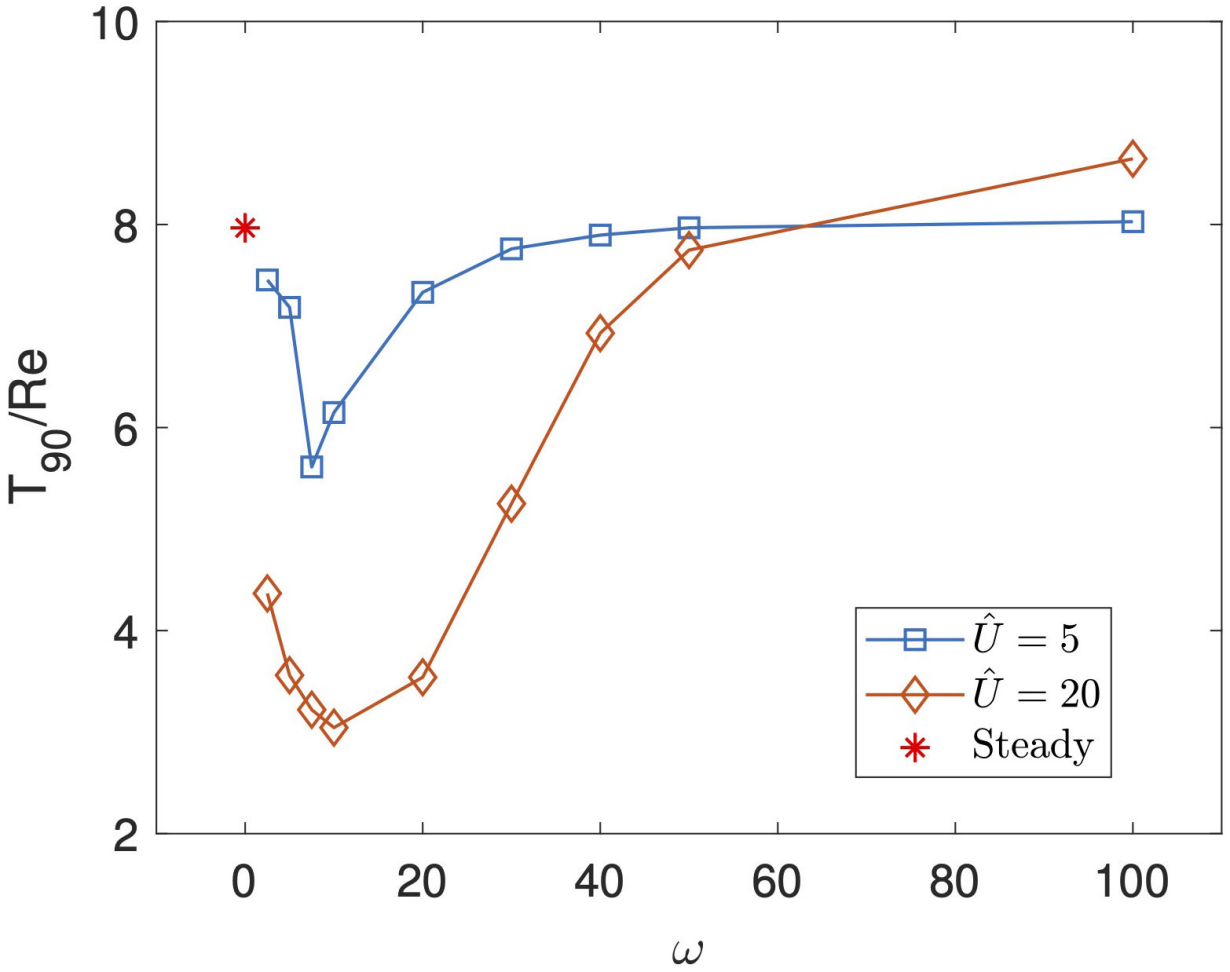
$T_{90}/Re$ as a function of ω for $\hat{U}=5$ and $\hat{U}=20$ in blue and orange respectively, with Re=50 and Sc=10 . The red star indicates the value of $T_{90}/Re$ for a steady parabolic inlet flow.

Taking the frequency analysis further, we explore a wider range of frequencies in **Figure 5**, in which we present $T_{90}/Re$ over a range of frequencies ω , and for two amplitudes of $\hat{U}=5,20$ in blue and orange respectively. The $T_{90}/Re$ value for a steady inlet flow is also shown in red for comparison purposes. We see that optimal wash-out times occur for a frequency around ω=10 . As the frequency is decreased towards zero, the wash-out times tend seem to be approaching the steady value which corresponds to a system with ω=0 . As the frequency increases from ω=10 , the wash-out times increase, with the high frequency wash-out times again becoming more similar to the steady system. This may be understood by the observation that at a high frequency the large, central fluid vortex in the cavity remains mostly undisturbed, and thus wash out is similar to the steady scenario.

**Figure 6:**
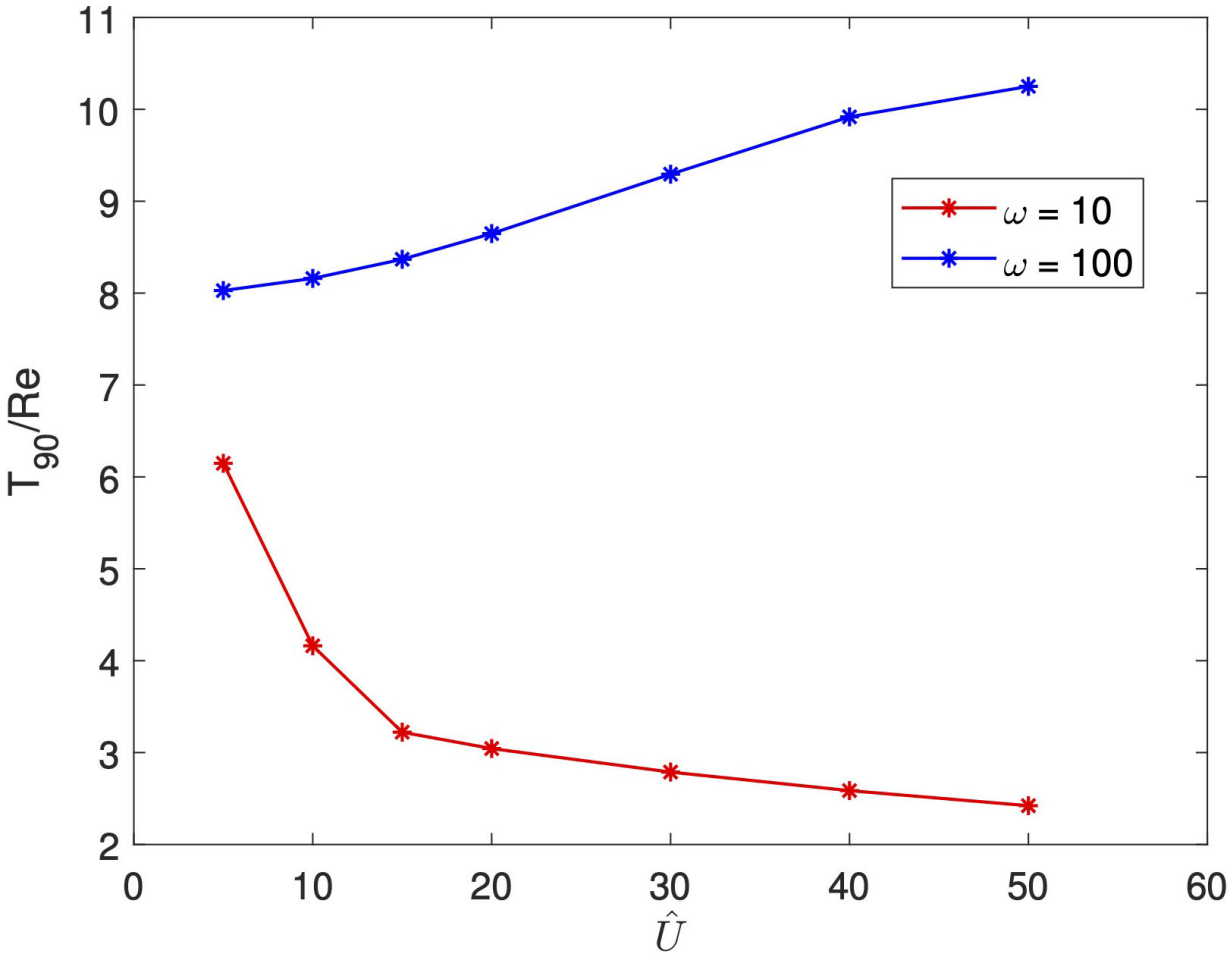
$T_{90}$ for increasing $\hat{U}$ , for Re=50 , Sc=10 and ω=10,100 .

As **Figure 4** was conducted for a fixed amplitude, we now address the question of whether a larger amplitude (which induces both increased vortex size and increased vortex disruption) will result in decreased wash-out times. To determine the impact of the oscillations amplitude, in **Figure 6** we plot wash-out time $T_{90}/Re$ for increasing amplitude $\hat{U}$ , for a low and high frequency. We see an intriguing feature. In the high frequency case, wash-out time increases with amplitude. In the low frequency case, though, we see the reverse: wash-out time decreases with amplitude. This highlights the delicate balance of the competing effects of increased vortex size and increased vortex disturbance on wash-out, and the strong dependence of wash-out on frequency. For the high frequency case, evidently the increasing vortical size outweighs the increasing vortical disruption that occurs with increasing amplitude, while at low frequency, the opposite occurs, with increasing vortical disruption outweighing the increasing vortical size. Note the nearly 10-fold difference in wash-out time at large amplitude; we contrast this with the relatively small difference (approximately 10%) in ΘRe at the same amplitude (**Figure 2**). We conclude that small changes in vortex disruption can have a significant impact on debris wash-out.

### 3.3 Comparison to the leading industry method

We have shown that under the right conditions an oscillatory cavity inlet flow can disturb vortices within the cavity and reduce wash-out times. A similar technique is currently employed in clinical practice to remove kidney stone dust. The process involves delivering an extra bolus of fluid into the cavity to wash away any excess dust that may be obscuring the view of the camera. In the hanging saline bag set-up, a bolus of fluid is delivered by squeezing the bag to introduce more upstream pressure and hence a brief increase in flow rate. This technique is known as flushing. An electronic pump may replicate the flushing process by increasing the flow rate for a short period of time. Due to the nature of how the flush is administered, the magnitude of the flush can be quite variable. Instead, using an electronic pump to control the delivery of a flush opens the possibility to have finer control over the magnitude and duration of the flush, as well as the time at which it is administered. Therefore, it is of interest to determine how varying these quantities impacts the wash-out time. We further compare wash-out times under the flushing procedure to our oscillatory flow setup.

To simulate the flushing process, we consider an initially steady inflow of fluid such that, at a given time, the inlet flow rate is increased in a step-wise fashion, for a fixed duration before returning to the baseline value.

To this end, we replace the inlet flow condition with


(18) $$u=\left(\left(1+\frac{k}{Re}\right)\left(1-y^{2}\right),0\right){\text{ on Γ}}_{in}\;,$$

where k is given as


(19-21) $$k=\{\begin{array}{ll} 0 & t<F_{start}Re\\ \kappa & F_{start}Re\leq t<F_{stop}Re\\ 0 & t>F_{stop}Re. \end{array}$$

Here κ/Re is the fractional increase in inlet flow rate, and the flush is applied between $F_{start}Re$ and $F_{stop}Re$ . In each case presented below, the numerical solver terminates once the value of $T_{90}$ has been reached, which may occur before $t=F_{stop}Re$ is reached.

**Figure 7:**
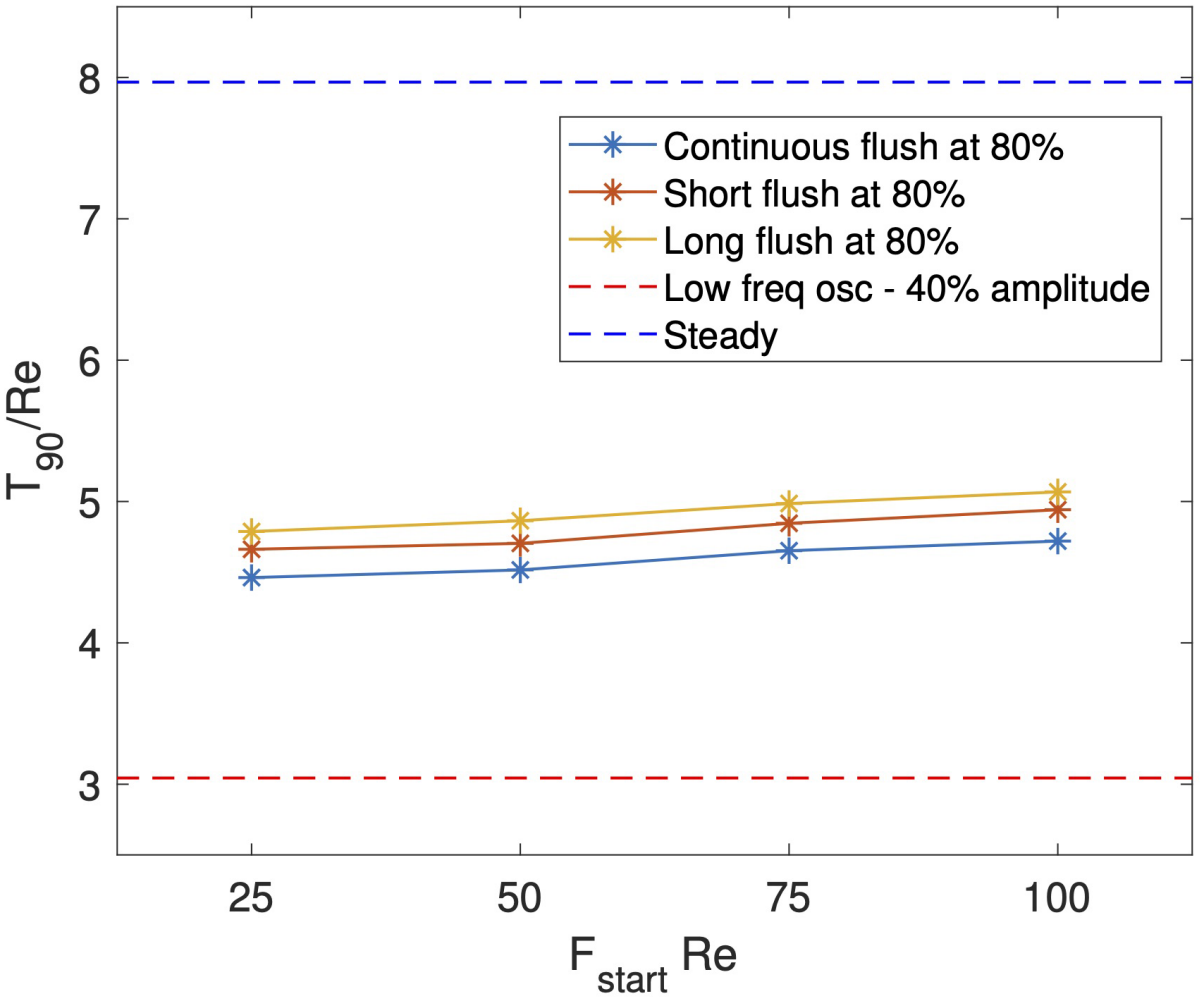
Comparison of $T_{90}/Re$ for a range of inlet conditions: steady flow (blue dashed), oscillatory (red dashed), and discrete flushes of continual (blue solid), short (red solid), and long (orange solid) duration.

As defined here, flushing consists of 3 independent parameters: κ , $F_{start}$ , and $F_{stop}$ . We anticipate that the sudden increase and subsequent decrease in flow rate creates a vortex disturbance in a similar manner to the oscillatory inlet. To investigate the impact of the start and stop times on wash-out reduction, we begin by fixing the flushing magnitude and varying the time at which the flush is administered for three scenarios, outlined below and illustrated in **Figure 7**. The light blue points correspond to the case of a continuous flush which once started does not stop. The second and third cases, given in orange and yellow respectively, are flushes of a specified duration. Explicitly, $F_{stop}Re=F_{start}Re+25$ for the short flush and $F_{stop}Re=F_{start}Re+75$ for the long flush. In both cases, κ/Re=0.8 , to allow comparison with an oscillatory inlet with an amplitude of 40%. In **Figure 7**, we also give the wash-out times for both the steady inlet flow and oscillatory inlet flow with ω=10 and $\hat{U}/Re=0.4$ , in dark blue and red dashed, horizontal lines respectively. We see that all cases of flushing provide shorter (approximately 50% reduction) wash-out times compared to the steady scenario. The later the flush is administered, the less impact it has on reducing wash-out time, although the difference is small compared to the steady scenario. A short flush performs better than a long flush, suggesting that the closer the start and end times are together the greater the impact of dust wash-out. However, the benefit of a sustained increase in inlet flow in the continual flush scenario out-performs the added benefit of a second vortex disturbance event generated from stopping the flush, as shorter wash-out times are observed in the continuous flush case. These results highlight the benefit of flushing compared to not flushing, but for all flush cases considered, wash-out times were comparable. However, the case of sustained oscillatory flow had a significantly shorter wash-out time than all flushing considered.

**Figure 8:**
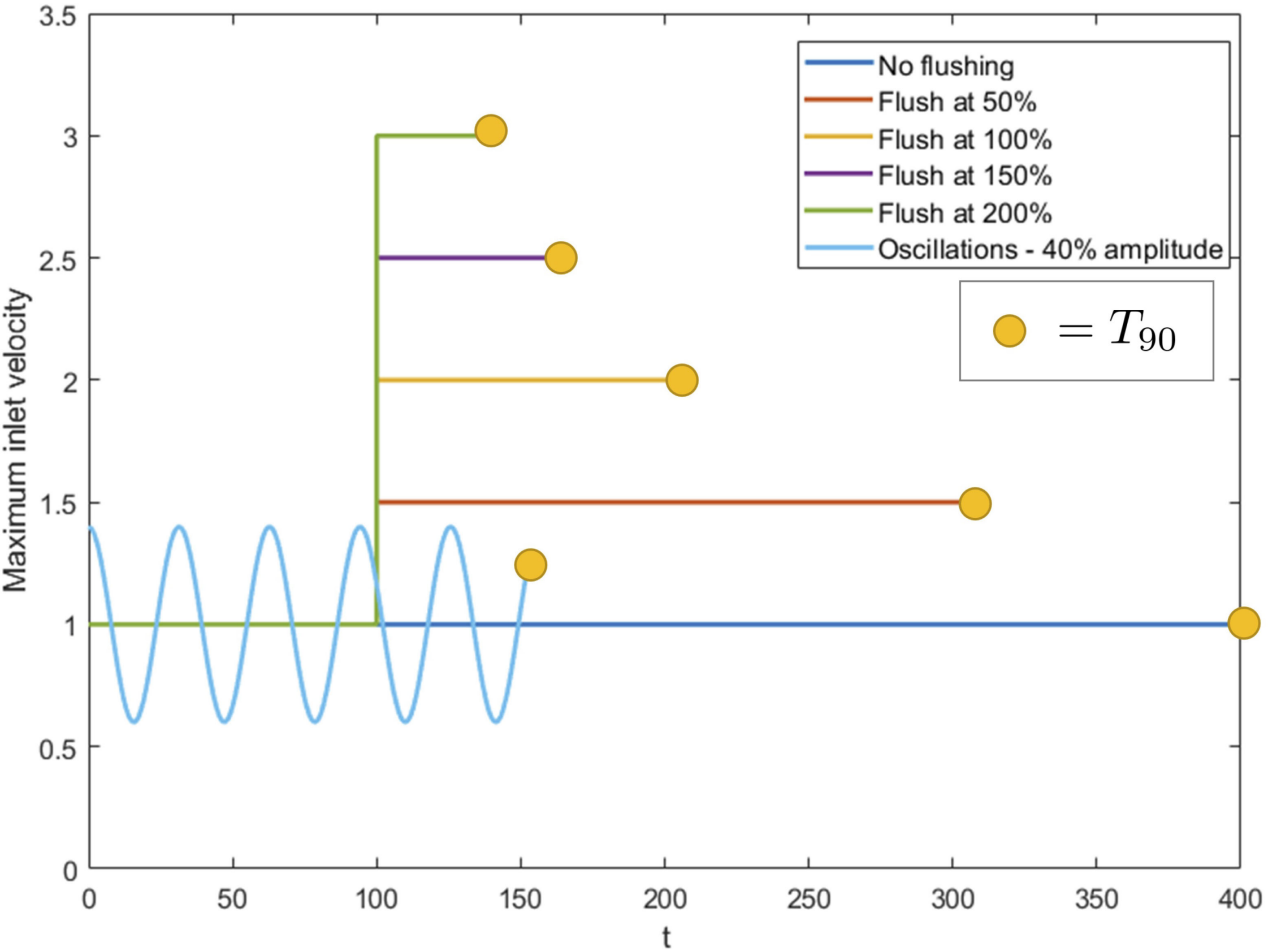
Maximum inlet velocity over time for increasing flush power compared with an oscillating inlet flow with ω=10 and $\hat{U}=20$ , for Re=50 and Sc=10 . Each case stops once the $T_{90}$ value has been reached, indicated by the yellow markers.

In **Figure 8** we plot the maximum inlet velocity over time for increasing flush magnitude κ/Re×100 varying from 0−200% and an oscillatory inlet with ω=10 and $\hat{U}/Re=0.4$ . Each case stops when $T_{90}$ is reached. The base maximum inlet flow and time at which the flush is introduced is the same for all cases. We see that as we increase the flush magnitude, the dust is washed away faster. The relationship appears to be non-linear, suggesting less of an improvement for ever increasing flush magnitude. While these results show that a larger flush is better for removing dust, it may have additional negative effects to the cavity such as introducing higher shear stresses on the kidney walls and increasing cavity pressures. Comparing these results to the best case oscillatory scenario, we see similar wash-out times to the 200% flush, though the oscillatory case achieves this with a much smaller maximum inlet velocity.

### 3.4 Extension to different domains

Up to now we have considered an idealised two-dimensional geometry of the kidney calyx, which we shall refer to as *kideal*. Relaxing the assumption of a two-dimensional cavity and exploring a three-dimensional geometry is beyond the scope of this paper (see (20) for 3D modelling work on fluid flow in the kidney). In this rectangular geometry, we have demonstrated a clear improvement in wash-out time under high-amplitude, low frequency oscillatory inlet flow. The question remains of whether this result is particular to this geometry or more robust to changes in domain geometry. To investigate, we consider six different cavity geometries, five of which are extensions of the previous rectangular domain, and a sixth based on a more realistic (but still 2D) model of a full kidney cavity. We first aim to establish whether the same qualitative behaviour of fluid flow exists within each new domain. Further to this, we evaluate the wash-out time in two scenarios for each domain: the first with a steady parabolic inlet and the second with an unsteady, oscillatory inlet. For simplicity, we set the flow parameters based on our previous analysis, and focus on a single oscillatory case of low frequency and high amplitude inlet flow.

**Figure 9:**
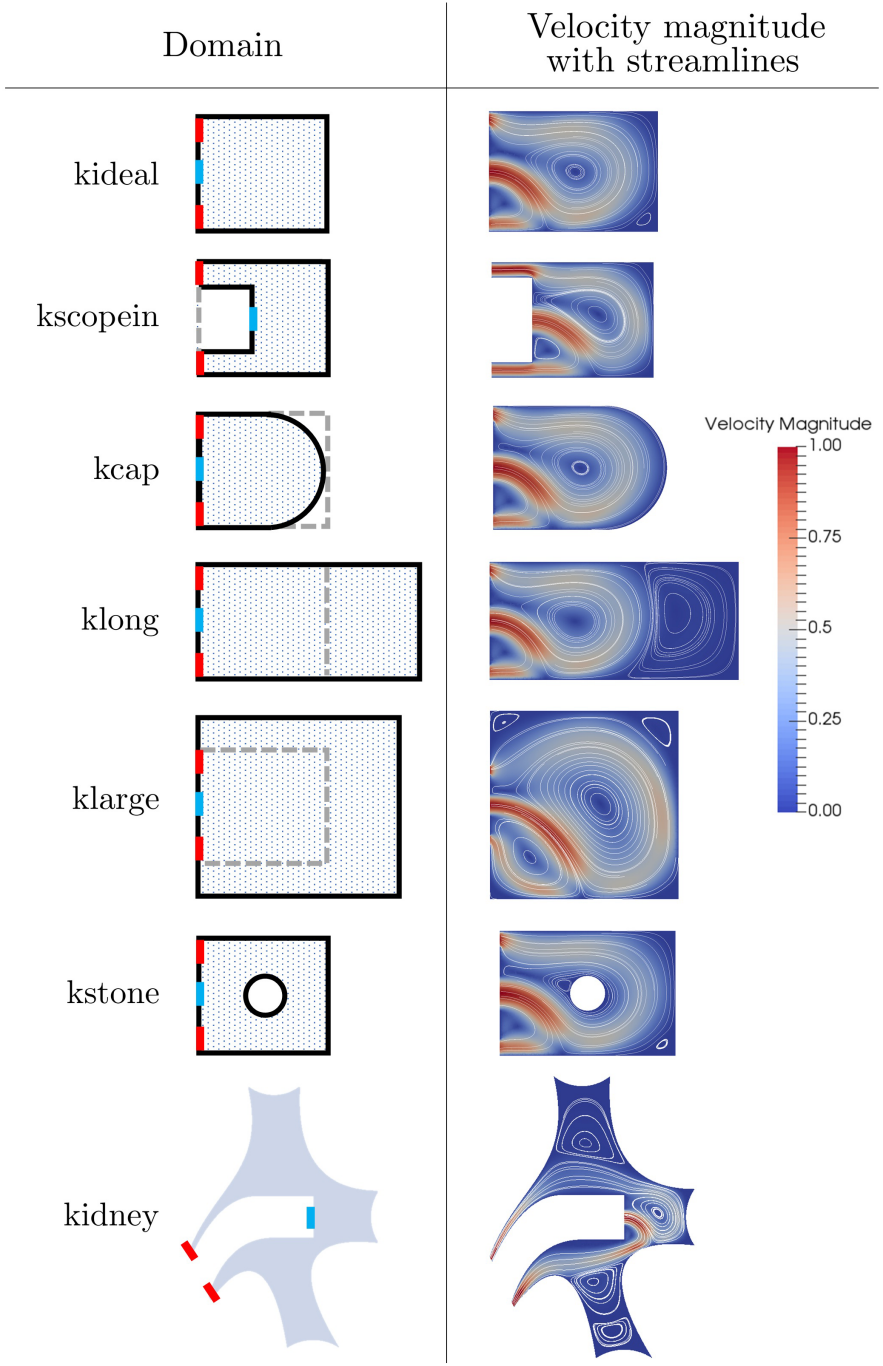
The seven cavity geometries domains along with their steady solutions for fluid velocity magnitude with streamlines. The domain inlets and outlets are marked as blue and red respectively.

In **Figure 9** we present streamline and velocity magnitudes in the case of steady flow for each of the seven domains considered.

On each domain sketch (apart from *kidney*) the original geometry *kideal* is shown in grey for comparison, with the inlet shown in blue and the outlets shown in red. In all cases, we see a qualitatively similar fluid structure characterised by a large central vortex. In particular, we highlight that even in the case of a geometry representative of the full kidney cavity (case 7), with multiple calyces present, the additional calyces have very minimal impact on the central vortex in the target calyx and the flow structure.

We now turn to the wash-out times for each scenario. The steady and oscillatory $T_{90}$ value for each case is given in **Table 1**. The results are generated for Re=50 , Sc=10 , ω=10 , and $\hat{U}=20$ . In all cases an oscillatory inlet improves wash-out times, with most times being at least halved. The best improvement was seen in *klarge*, due to the central vortex being larger and thus trapping more dust. When a disruption effect is introduced here a nearly 75% reduction in wash-out is obtained with oscillatory inlet. For *kscopein* we consider the notion of a ureteroscope that has been pushed slightly further towards the back of the cavity to aid in washing away excess debris. Note that as the scope is inserted further in and the cavity length is reduced, we reach a point at which the solution structure returns to being symmetrical and the central vortex no longer exists (result not shown here). This feature of the dependence of critical Reynolds number above which asymmetrical solutions on cavity length was shown in (26). Improvement in wash-out time is slightly reduced in *klong* due to the presence of a second vortex forming at the back of the cavity. The oscillations failed to aid in disturbing the additional vortex, and so dust that was trapped could only exit the cavity on the slower diffusive timescale. The smallest improvement was seen in *kstone*. Here, a circular cut-out was removed from the domain, representing a solid stone situated in the centre of the cavity. The smaller improvement in wash-out can be explained by the fact that the centre of the vortex has been blocked, meaning not much dust is trapped in the first place. Introducing the disruption effect in this case does improve wash-out, but to a lesser extent.

**Table 1:** The steady and oscillatory *T*_90_ results for each shape considered, along with a percentage difference from the steady case.

Shape	Steady *T*_90_	Oscillatory *T*_90_	Percentage difference
kideal	400.47	145.84	63.58%
kscopein	213.99	88.5	58.64%
kcap	323.37	107.69	66.70%
klong	2652.95	1806.58	31.90%
klarge	2796.85	704.59	74.81%
kstone	242.14	189.12	21.90%
kidney*	903.23	315.12	65.11%

In the illustrative example *kidney*, the dust does not initially occupy the entire domain, instead only occupying the right-most calyx to which the scope is pointed. We find that the dust is washed away in the streamlines alongside the scope and out through the base outlets. Again, introducing oscillations to this domain gives a significant improvement of 65% to wash-out time.

We conclude that the vortex characteristics seen in the idealised domain extend to the other six geometries, and that oscillating the inlet flow at a low frequency and high amplitude improves wash-out times when comparing to a steady inlet for all geometries.

## 4 Summary and discussion

Efficient kidney stone dust removal is vital for an effective ureteroscopy procedure. Advances in fluid irrigation management systems in ureteroscopy have the potential to improve dust wash-out times through the control of fluid delivery. By modelling the irrigation fluid flow and kidney stone dust transport within the cavity, in this paper we have gained insight into the mechanisms by which time dependent fluid delivery may be harnessed to facilitate debris clearance.

We have modelled the fluid flow and dust transport in a rectangular, two dimensional cavity, driven by an oscillatory, parabolic inlet flow. Here we have focussed on a flow regime dominated by a large central vortex in which dust has the potential to become trapped, leading to long wash-out times. We first analysed the impact of frequency and amplitude on the vortex characteristics, demonstrating that while the time-averaged size of the vortical regions increases with amplitude, largely insensitive to frequency, the variation in size of vortical regions increases with amplitude, and importantly is higher in the case of low frequency. To investigate how these combined effects impact the wash-out, we solved the advection-diffusion equation for dust concentration. Here, we demonstrated the intriguing feature that wash-out time increased with amplitude in the high-frequency case, but decreased with amplitude in the low-frequency case. Thus, we have uncovered the key conclusion that wash-out time can be significantly decreased by imposing a high-amplitude, low-frequency oscillatory inlet flow. This result was shown to be robust to changes in geometry, and also compared favourably to the industry standard method of a discrete flushing event. However, it is important to emphasise that we have used a Schmidt number that vastly overestimates diffusive effects and is completely unrealistic for kidney stone dust. This choice was made for computational purposes, enabling the theoretical model to compute dust washout in cases with minimal vortical disruption. While we acknowledge that this choice is unphysical, nevertheless our results should qualitatively hold at larger Schmidt numbers, and indeed the difference in washout time between the low frequency and high frequency inlets only becomes more pronounced with increasing Sc (see **Supplementary Material section**). Thus, our main thesis – that vortical disruption through oscillatory flow is beneficial in dust washout – becomes only more relevant as dust diffusivity is diminished. A more realistic quantitative comparison on the benefit of oscillatory flow to dust washout may be attained by using a much larger Schmidt number. An alternative metric to $T_{90}$ would be required to simulate this, though, as the hugely increased computation time in the high frequency case would render the system unsolvable on any reasonable computational timescale. On the other hand, in cases of minimal vortical disruption, in which a significant portion of dust is trapped within closed streamlines, we can estimate the timescale for dust washout based on the diffusive timescale, which as shown in **Supplementary Material section** is $O\left(10^{7}\right)$ s! Clearly, vortical disruption by some means is critical for dust removal.

An obvious direction for future work is extension to a three dimensional cavity. Recent work has considered cavity flow in 3D (20), with steady inlet conditions and stone particles large enough to impact the flow; in this regime the stone particles themselves can disrupt vortices. It remains to consider oscillatory flow and vortical disruption with small dust particles in a realistic 3D kidney geometry. However, this comes with a very high computational cost, requiring solving the unsteady Navier-Stokes and advection diffusion equations in a three dimensional domain. While such an extension would be valuable in confirming our results, the reduced computation time and relative simplicity in terms of flow analysis that a 2D geometry affords underscores the utility of idealised modelling with simplified geometries. Indeed, we have shown the potential value of an oscillatory inlet flow in dust removal in ureteroscopy, and have uncovered mechanisms that, with further computational interrogation in a more realistic geometric setting, have strong potential for clinical translation.

## Data availability statement

The original contributions presented in the study are included in the article/**Supplementary Material**. Further inquiries can be directed to the corresponding author.

## Author contributions

DM, BT, and SW designed the research, HR conducted the analysis. DM, SW, and HR all contributed to the writing of the paper. All authors contributed to the article and approved the submitted version.

## Footnotes

1 The situation is different for larger size stone fragments, in which case the fluid flow and stone fragments motion are fully coupled; this scenario is modelled in the recent work (19), though under steady flow conditions.
