# Supplementary material for: Harnessing oscillatory fluid behaviour to improve debris wash-out in ureteroscopy
Source: Front Urol. 2023 Aug 30;3:1182919. doi: 10.3389/fruro.2023.1182919 (PMC12327291; doi:10.3389/fruro.2023.1182919)
Supplement: Supplementary file 1 [file DataSheet_1.pdf]

Harnessing oscillatory fluid behaviour to improve debris wash-out in ureteroscopy – Appendix

H.C.A. Reynolds [1], **B.W. Turney** [2], **S.L. Waters** [1] **and D.E. Moulton** [1,*]

[1] *Mathematical Institute, University of Oxford, Oxford, UK,* [2] *Nuffield Department of Surgical Sciences, University of Oxford, Oxford, UK*

Correspondence*:
DE Moulton, Mathematical Institute, University of Oxford, Oxford OX2 6GG, UK
moulton@maths.ox.ac.uk

1 STEADY SYSTEM

This appendix outlines the steady system which is solved to give an initial condition for the unsteady system. In order to be a valid initial condition for the unsteady problem, the steady problem uses the same domain as laid out in the problem description. We non-dimensionalise the problem via the same scalings as given in main text equation (3).

The non-dimensional, steady Navier-Stokes equations which govern the fluid is then given by

$$Re\left(\left(\boldsymbol{u}_{steady}\cdot\nabla\right)\boldsymbol{u}_{steady}\right) = -\nabla p_{steady} + \nabla^2\boldsymbol{u}_{steady}\ , \tag{1}$$

$$\nabla\cdot\boldsymbol{u} = 0\ . \tag{2}$$

At the inlet to the cavity we prescribe fully-developed parabolic flow, given by

$$\boldsymbol{u}_{steady} = \left(\left(1-y^2\right), 0\right) \quad \text{on} \quad \Gamma_{in}\ , \tag{3}$$

At the outlet boundaries we prescribe zero stress such that

$$\boldsymbol{\sigma}\cdot\boldsymbol{n} = \boldsymbol{0} \quad \text{on} \quad \Gamma_{out}\ . \tag{4}$$

We impose no-slip on all impermeable walls, given by

$$\boldsymbol{u}_{steady} = (0,\ 0) \quad \text{on} \quad \Gamma_{walls}\ . \tag{5}$$

We solve the system numerically via a finite element formulation, which is implemented using the open source finite element library Firedrake [7]. To solve, we use a stationary incompressible Navier-Stokes solver with Reynolds robust pre-conditioner [3]. This solution is used in main text equation (9) as the initial condition to the unsteady system, as well as for comparison purposes to the unsteady results.

2 ON PARAMETERS AND DIMENSIONLESS CONSTANTS

Table 1 provides a list of parameter values for the fluid properties, length scales for kidney calyx cavity and scope radius, typical fluid flux in ureteroscopy, and dust particle radius. Standard parameter values for the

Description	Parameter	Value	Units	Reference
Working channel radius	a	0.06	cm	[12]
Cavity length	l_c	1	cm	[2]
Fluid density	ρ^*	1	g/cm^3	-
Fluid viscosity	μ^*	0.01	g/cm s	-
Mean fluid velocity	$\bar{U}^*$	8 to 350	cm/s	[12, 11]
Fluid flux	Q	0.5 to 2	cm^3/s	[10]
Dust particle radius	R	0.001 to 0.1	cm	[5]

Table 1. System parameter values.

viscosity and density of a fluid similar to water have been taken. From these, we may compute both the Schmidt number and Reynolds number.

Schmidt number

An estimate for the Schmidt number can be obtained via the Stokes-Einstein equation [1, 6]

$$Sc = \frac{\nu}{D} = \frac{(\mu/\rho)}{k_B T/\left(6\pi\mu R\right)} = \frac{6\pi\mu^2}{k_B T\rho} R\,, \tag{6}$$

where R is the radius of a spherical particle, k_B is the Boltzmann constant, T is temperature, and R is spherical radius of the particles. Taking $T = 310$K, and the fluid parameters as given in Table 1, gives $Sc = 2.13 \times 10^{12} R/\mathrm{m}$. Dust particle size varies significantly based on the details of the laser and approach of the operating clinician [4]. Studies on dust size have found that Holmium:YAG laser lithotripsy generates particles ranging from $\leq$63 μm to <2 mm [5]. Stone dust was defined as having an upper limit of about 250 μm. Given the assumption in our model that the dust does not impact the flow, our work is really only relevant for small dust particles. But even for very small dust particles on the order of 10 μm, the Schmidt number is in excess of 2×10^7, which corresponds to a diffusion coefficient $D \sim 10^{-9}$ cm^2/s. Considering a length scale $L \sim 0.1$ cm (approximate size of a relatively small vortex in the channel), this gives a diffusive timescale

$$T_{\text{diff}} = \frac{L^2}{D} \sim 10^7 \text{ s}.$$

This time-scale T_{diff} reflects the slower of two processes for diffusion of a tracer across closed streamlines, the other being shear-augmented diffusion [9], but T_{diff} is the rate-limiting timescale for dust particles to diffuse out of a vortex.

For these values, diffusion is effectively negligible, and the system is almost entirely driven by advection. It would, accordingly, be very accurate to model the system without diffusion. However, if diffusion is neglected altogether, particles may become permanently trapped inside vortices. In the limit of steady flow, for instance, any dust particles contained inside a closed streamline will never escape the cavity. If more than 10% of the dust is trapped in this way, the washout time T_{90} diverges to infinity. As the high frequency case behaves similarly to the case of steady flow, computation time becomes very significant.

This is demonstrated in Figure 1 and Table 2. Figure 1 plots the washout time T_{90}/Re for both low and high inlet frequency ($\omega = 10$ and $\omega = 100$), and for Sc taking the values 1, 10, and 100. In the low frequency case, since the vortex is significantly disrupted, the dust washes out on the advective timescale, and the washout time is barely impacted by diffusion. For the high frequency case, on the other hand, vortical disruption is minimal and insufficient to free trapped dust; therefore washout relies on diffusion of trapped dust, and accordingly T_{90} rises significantly with Sc. Table 2 gives the computation time associated

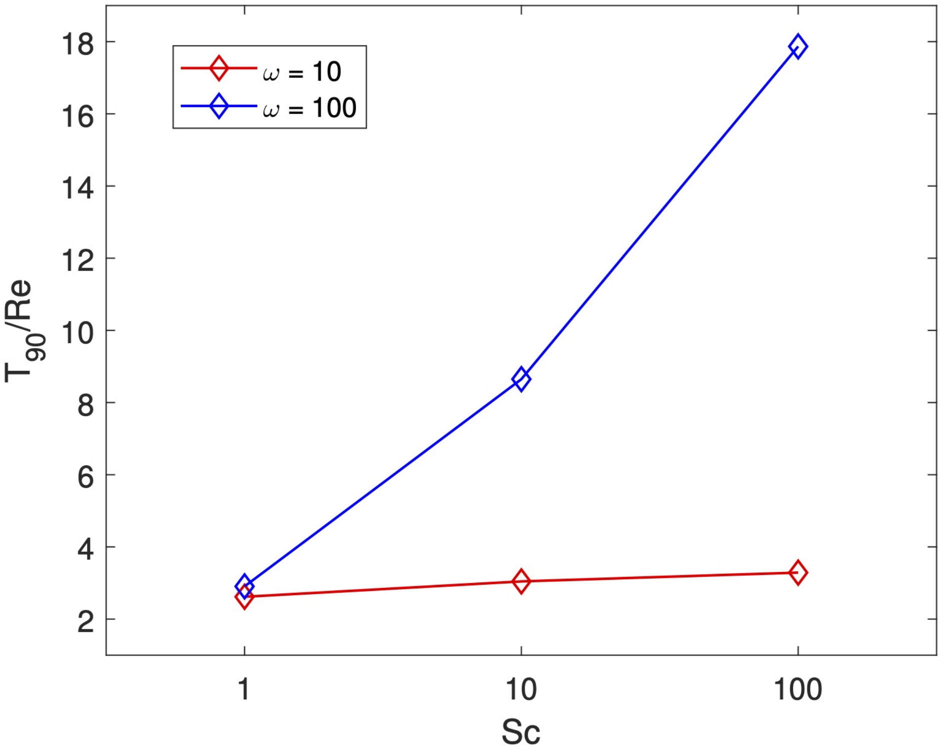

Figure 1. T_{90}/Re for increasing Schmidt number Sc, for $Re = 50$, $\hat{U} = 20$, and $\omega = 10, 100$.

Schmidt number	ω	Computation time (minutes)
1	10	56.05
10	10	64.04
100	10	68.74
1	100	64.65
10	100	192.5
100	100	610.2

Table 2. Computational time in minutes of the results seen in Figure 1.

with the points appearing in Figure 1; this reflects the same trend. Extrapolating these curves and the computation time to larger Sc shows the impracticality of simulating at physically relevant values for stone dust.

On the one hand, this observation reinforces the key hypothesis of our analysis that there is a benefit to disturbing vortices with oscillatory flow. On the other hand, having advection only is probably not entirely accurate for the ureteroscopy application. As well as having some very small degree of diffusion, particles will also settle under gravity, and/or move due to some initial momentum obtained during fragmentation from the kidney stone. One obvious extension to our work would be to include gravitational forces. We have opted not to do so here, as there is not a consistent means of including gravity in our 2D model. That is, the direction of gravity is unlikely to lie in the plane of simulation, and in any case the direction of gravity relative to the channel would vary significantly based on the particular kidney calyx and the positioning of the patient. Accordingly, it is likely that we are underestimating washout times, and in some cases perhaps vastly underestimating washout times. Nevertheless, the qualitative nature of our results is

Reynolds number	ω/Re	Computation time (minutes)
50	0.2	57.08
100	0.2	165.27
50	2	169.1
100	2	425.65

Table 3. Computational time in minutes of the results seen in Figure 2.

likely to translate to a system with no diffusion, where indeed the benefit of oscillatory flow will be much stronger.

Reynolds number

The Reynolds number is defined as

$$Re = \frac{\rho U L}{\mu},$$

where U is a characteristic velocity, L a characteristic length scale, and ρ and μ are respectively the density and dynamic viscosity. Two natural length scales in the system are the working channel radius, which defines the inlet flow profile, and the cavity length; we have used the former in defining Re in the main text. Obtaining a reasonable velocity scale is less straightforward. In a typical ureteroscopy procedure, irrigation fluid is delivered to the kidney with a flux ranging from $Q \sim 0.5$ cm^3/s to $Q \sim 2$ cm^3/s. Supposing a parabolic unidirectional flow profile through a circular channel of radius $a = 0.06$ cm, the average velocity is

$$U = \frac{4Q}{3\pi a^2},$$

which ranges from 60 to 230 cm/s which, using the fluid parameter values in Table 1, gives a Reynolds number in the range $350 \lesssim Re \lesssim 1400$. Though note that inserting a working tool can significantly affect both the flux and the velocity [12], so that with a tool inserted the velocity may drop as low as 40 cm/s, giving $Re \sim 240$. However, these values are really only appropriate for 3D cavity flow. In the 2D system, we have used a smaller value of $Re = 50$. This is partly motivated for comparison with previous studies [12, 11] which used a similar range of Re in a very similar geometry. In particular, [11] conducted experiments in a rectangular cavity designed to mimic 2D flow and with similar parameter values as utilised here – in that work the Reynolds was computed to be $Re \sim 40$. Similar to the choice of Schmidt number described above, our choice of Reynolds number is also partly motivated by computational considerations. As noted in the main text, the flow bifurcation from symmetric flow to an asymmetric flow pattern with central vortex occurs around $Re = 20$. As Re increases, the asymmetric flow persists, and the central vortex grows in size [8]. For increasing Re, there is an increasingly large region of closed streamlines, in which dust can become trapped. As in the case of large Sc, this reinforces the necessity of disturbing vortices, e.g. via oscillatory flow as we propose, but again there is an increasing computational cost. Fig 2 plots the T_{90}/Re for $Re = 50$ and $Re = 100$ in both the low frequency ($\omega = 10$) and high frequency ($\omega = 100$) cases, while Table 3 gives the corresponding computation time for each run. [1] As with increasing Sc, increasing Re increases the computation time, with the increase in central vortex size leading to comparative increases for both low and high frequency.

[1] Recall that as we have scaled time with the velocity scale, the appropriate comparative dimensionless washout time is T_{90}/Re. Also, given that the amplitude and frequency are scaled on Re (see main text equation (6)), in simulating with increased Re, we have proportionally increased ω and $\hat{U}$ so that ω/Re and $\hat{U}/Re$ are held constant.

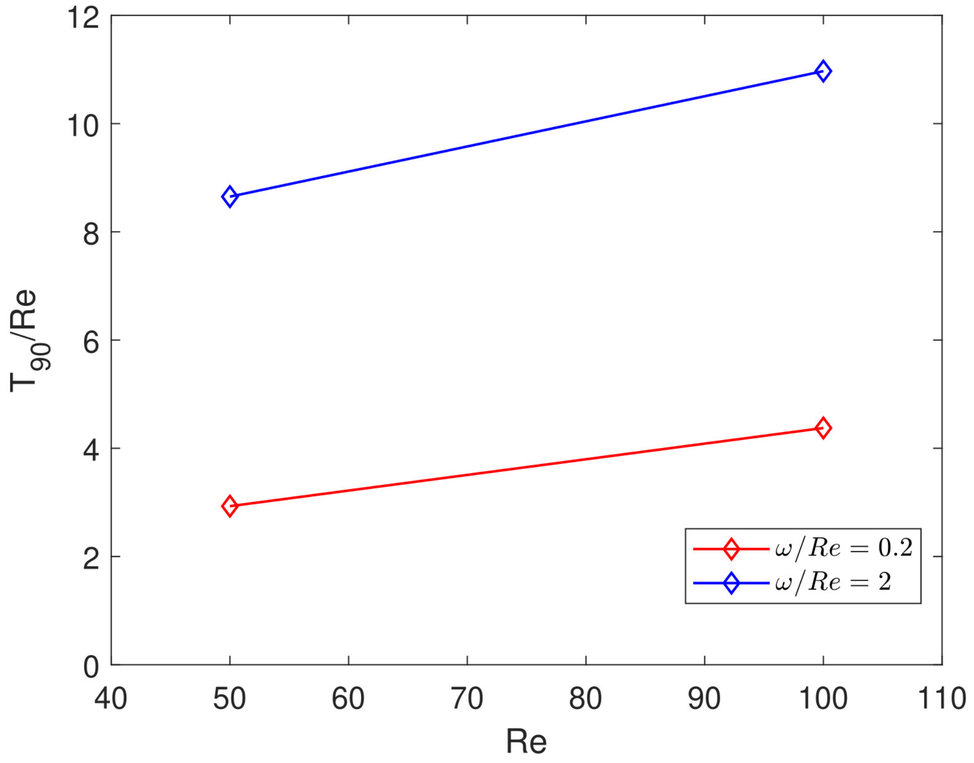

Figure 2. T_{90}/Re for increasing Reynolds number Re, for $Sc = 10$, $\hat{U}/Re = 0.4$, and $\omega/Re = 0.2, 2$.

REFERENCES

[1]A Einstein. Elementare theorie der brownschen) bewegung. *Zeitschrift für Elektrochemie und angewandte physikalische Chemie*, 14(17):235–239, 1908.

[2]SA Emamian, MB Nielsen, JF Pedersen, and L Ytte. Sonographic evaluation of renal appearance in 665 adult volunteers: correlation with age and obesity. *Acta Radiologica*, 34(5):482–485, 1993.

[3]PE Farrell, L Mitchell, and F Wechsung. An augmented lagrangian pre-conditioner for the 3d stationary incompressible navier–stokes equations at high reynolds number. *SIAM Journal on Scientific Computing*, 41(5):A3073–A3096, 2019.

[4]LA Hardy, V Vinnichenko, and NM Fried. High power holmium: Yag versus thulium fiber laser treatment of kidney stones in dusting mode: ablation rate and fragment size studies. *Lasers in surgery and medicine*, 51(6):522–530, 2019.

[5]EX Keller, V De Coninck, S Doizi, M Daudon, and O Traxer. What is the exact definition of stone dust? an in vitro evaluation. *World Journal of Urology*, 39:187–194, 2021.

[6]AL Kholodenko and JF Douglas. Generalized stokes-einstein equation for spherical particle suspensions. *Physical Review E*, 51(2):1081, 1995.

[7]F Rathgeber, DA Ham, L Mitchell, M Lange, F Luporini, ATT McRae, GT Bercea, GR Markall, and PHJ Kelly. Firedrake: automating the finite element method by composing abstractions. *ACM Transactions on Mathematical Software (TOMS)*, 43(3):24, 2017.

[8]HCA Reynolds. *Mathematical modelling of unsteady flows during ureteroscopy*. PhD thesis, University of Oxford, 2022.

[9]PB Rhines and WR Young. How rapidly is a passive scalar mixed within closed streamlines? *Journal of Fluid Mechanics*, 133:133–145, 1983.

[10]JG Williams. Mathematical modelling of fluid flows during ureteroscopic kidney stone removal. Chpt. 5, PhD Thesis, 2019.

[11]JG Williams, AA Castrejón-Pita, BW Turney, PE Farrell, SJ Tavener, DE Moulton, and SL Waters. Cavity flow characteristics and applications to kidney stone removal. *Journal of Fluid Mechanics*, 902, 2020.

[12]JG Williams, BW Turney, NP Rauniyar, TP Harrah, SL Waters, and DE Moulton. The fluid mechanics of ureteroscope irrigation. *Journal of endourology*, 33(1):28–34, 2019.
